# When Relief Backfires: A Case Report of Non-steroidal Anti-inflammatory Drug (NSAID)-Induced Acute Colitis

**DOI:** 10.7759/cureus.86992

**Published:** 2025-06-29

**Authors:** Bo Sun, Mina Hanna, Ravi Shekarrappa

**Affiliations:** 1 Family and Community Medicine, Trinity School of Medicine, Warner Robins, USA; 2 Medicine, Trinity School of Medicine, Warner Robins, USA; 3 Internal Medicine, Houston Healthcare, Warner Robins, USA

**Keywords:** acute gastrointestinal bleed, chronic daily headache, esophagogastroduodenoscopy (egd), gastric ulcer disease and nsaids and bleeding, ulcerative colitis (uc), colonoscopy

## Abstract

The use of non-steroidal anti-inflammatory drugs (NSAIDs) can be traced back to the Greco-Roman era, when willow tree bark extract was commonly used for its analgesic properties. Since their inception, NSAIDs have become a common component of treatment plans, with widespread use across various pathological conditions. However, there has been a recent increase in the incidence and prevalence of both upper and lower gastropathies. This case report examines an 18-year-old Caucasian male with no significant past medical history who presented with new-onset hematochezia and generalized fatigue. He reported daily over-the-counter NSAID use for more than two years prior to the onset of symptoms, primarily to self-treat debilitating daily headaches. Further evaluation with esophagogastroduodenoscopy (EGD) and colonoscopy with biopsy revealed colonic crypt infiltrates. He was diagnosed with acute colitis, discharged from our service, and advised to undergo a repeat colonoscopy in six weeks; however, the patient was ultimately lost to follow-up.

## Introduction

The first rendition of modern non-steroidal anti-inflammatory drugs (NSAIDs) was synthesized by Felix Hoffmann in 1897 in the form of acetylsalicylic acid, more commonly known as aspirin [1]. Today, NSAIDs are commercially available and routinely prescribed by clinicians worldwide for a wide range of conditions, making them among the most widely used medications globally. NSAIDs are known to be multi-faceted drugs, possessing analgesic, antipyretic, and anti-inflammatory properties. As a result of these characteristics, the Mayo Clinic reports that over 100 million NSAID prescriptions are filled annually in the United States [2,3]. With their widespread availability and versatility, NSAIDs have become a staple and often first-line therapy for conditions involving acute or chronic pain, headaches, arthropathies, and even for certain disease prevention strategies.

Though NSAIDs exist in various forms, their pharmacokinetics and mechanism of action are relatively consistent. Primarily, NSAIDs function by inhibiting the cyclooxygenase (COX) enzyme pathway, which ultimately halts the transformation of arachidonic acid into prostaglandins, prostacyclin, and thromboxane. These arachidonic acid derivatives are central to the biochemical cascades responsible for pain, fever, and platelet aggregation. By inhibiting COX enzymes, NSAIDs disrupt these pathways, thereby reducing pain, fever, and platelet clumping [4].

The COX enzyme exists in two isoforms: COX-1 and COX-2. Each isoenzyme exerts its effects in different parts of the body. In addition to various physiological functions, these enzymes play a key cytoprotective role in the GI mucosa by promoting the growth and maintenance of the mucosal barrier. They are also involved in mucus production and vasodilation, particularly during mucosal injury, which helps facilitate epithelial repair and regeneration. Consequently, inhibition of COX enzymes compromises the integrity of the GI mucosal barrier and renders it susceptible to damage [5].

Chronic COX inhibition has been associated with a spectrum of GI pathologies. Patients may present with nonspecific GI symptoms or more serious complications such as GI bleeding. In such cases, a relevant past medical history often reveals risk factors for GI bleeding. These may include, but are not limited to, age over 50, Reye syndrome, a history of complicated peptic ulcer disease or prior GI bleeding, smoking, heavy alcohol consumption, or chronic inflammatory/pain disorders requiring long-term NSAID use [6].

This case report examines a young male who initially presented with generalized fatigue, malaise, and melena, along with a significant history of chronic NSAID use to self-treat recurrent headaches. We aim to highlight an often-overlooked adverse effect of a commonly prescribed and frequently self-administered medication.

## Case presentation

An 18-year-old Caucasian male with no significant past medical history presented to the ED with a chief complaint of generalized weakness and malaise. The patient had recently been seen by his primary care physician for a routine follow-up and was found to have a decreased hemoglobin level of 7.9 g/dL (normal range: 13.2-16.6 g/dL) (Table 1). At that time, he reported ongoing hematochezia with most bowel movements for the past year. He also noted a significant increase in bowel movement frequency over the previous month, citing large amounts of bright red blood in each instance. He denied nocturnal bowel movements or changes in frequency. However, he did report generalized abdominal pain across all quadrants, described as a dull ache with an intensity of 2/10.

**Table 1:** Relevant laboratory values.

Values	Result	Normal Range
Hemoglobin (Hb)	7.5 g/dL	13.2-16.6 g/dL
Hematocrit (Hct)	27.90%	41-50%
Mean Corpuscular Volume (MCV)	61.9 fL	80-100 fL
Reticulocyte Count (Retic)	1.60%	0.5-2.5%
Prothrombin Time (PT)	10.8 s	11-13.5 s
Activated Partial Thromboplastin Time (aPTT)	27.1 s	21-35 s
Stool Occult Blood Test	Positive	-

His past medical and family histories were unremarkable, although he had undergone a tonsillectomy in early childhood. On admission to the ED, the patient stated that he was not taking any prescription medications, but he did report using over-the-counter (OTC) diphenhydramine for seasonal allergies and daily NSAIDs for headache management over the past three years.

On physical examination, the patient appeared pleasant, alert, and oriented to person, place, and time. He was in no acute distress. Abdominal examination was unremarkable. A rectal examination revealed maroon-colored stool in the rectal vault, prompting further evaluation.

The patient was admitted to the inpatient service for further workup. During his stay, he revealed that he had been experiencing recurrent daily headaches with a cap-like distribution for nearly three years. For symptomatic relief, he had been taking 600 mg of OTC NSAIDs twice daily since the onset of these headaches. He denied photophobia, phonophobia, nausea, or emesis.

At this point in care, he was assessed as having an unspecified gastrointestinal bleed, likely secondary to NSAID-induced peptic ulcer disease. He was also found to have microcytic anemia due to chronic GI blood loss and NSAID-induced rebound headaches. The patient was started on intravenous (IV) normal saline (0.9%) per hospital protocol, along with IV pantoprazole, a polyethylene glycol drip, oral acetaminophen for headache relief, and an iron sucrose infusion for anemia. An esophagogastroduodenoscopy (EGD) and colonoscopy with biopsy were ordered for further investigation.

**Figure 1:**
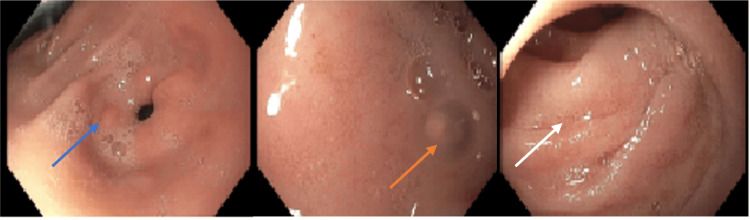
Esophagogastroduodenoscopy image showing a distal esophageal ulcer (blue arrow), non-bleeding gastric antral ulcers (orange arrows), and gastritis (white arrows). Image obtained with the patient’s expressed consent.

**Figure 2:**
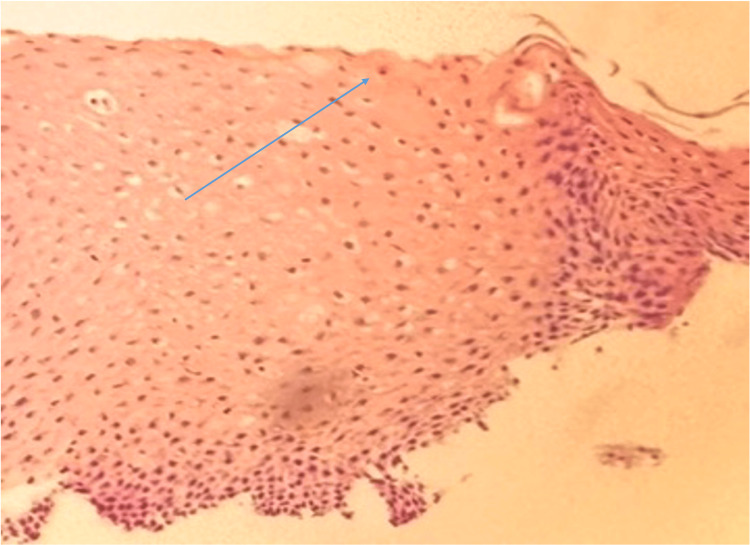
Biopsy of a gastroesophageal junction ulcer showing eosinophils (blue arrow) within the squamous epithelium. Image obtained with the patient’s expressed consent.

**Figure 3:**
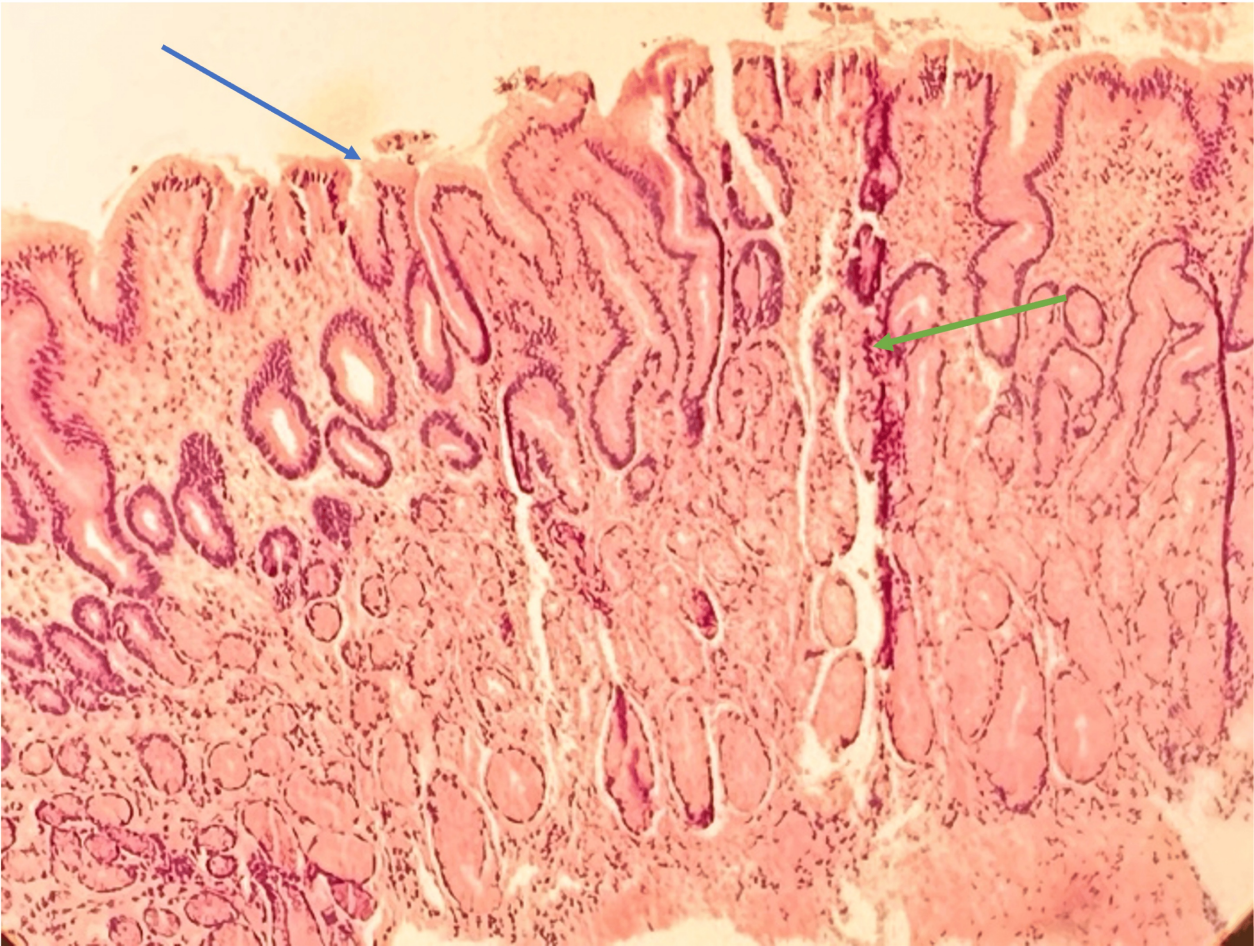
Biopsy of an antral ulcer showing edematous gastric mucosa (blue arrow), suggestive of chemical gastropathy, and corkscrew morphology (green arrow). Image obtained with the patient’s expressed consent.

EGD results revealed a possible distal esophageal ulcer and a non-bleeding gastric antral ulcer (Figure 1). Histopathological examination of biopsy specimens from the gastroesophageal junction showed edematous gastric mucosa with scattered lymphocytes, plasma cells, and eosinophils in the lamina propria (Figure 2). The gastric biopsy demonstrated elongated gastric pits with corkscrew morphology, suggestive of chemical gastropathy likely caused by chemical insult (Figure 3). No malignancy was observed.

**Figure 4:**
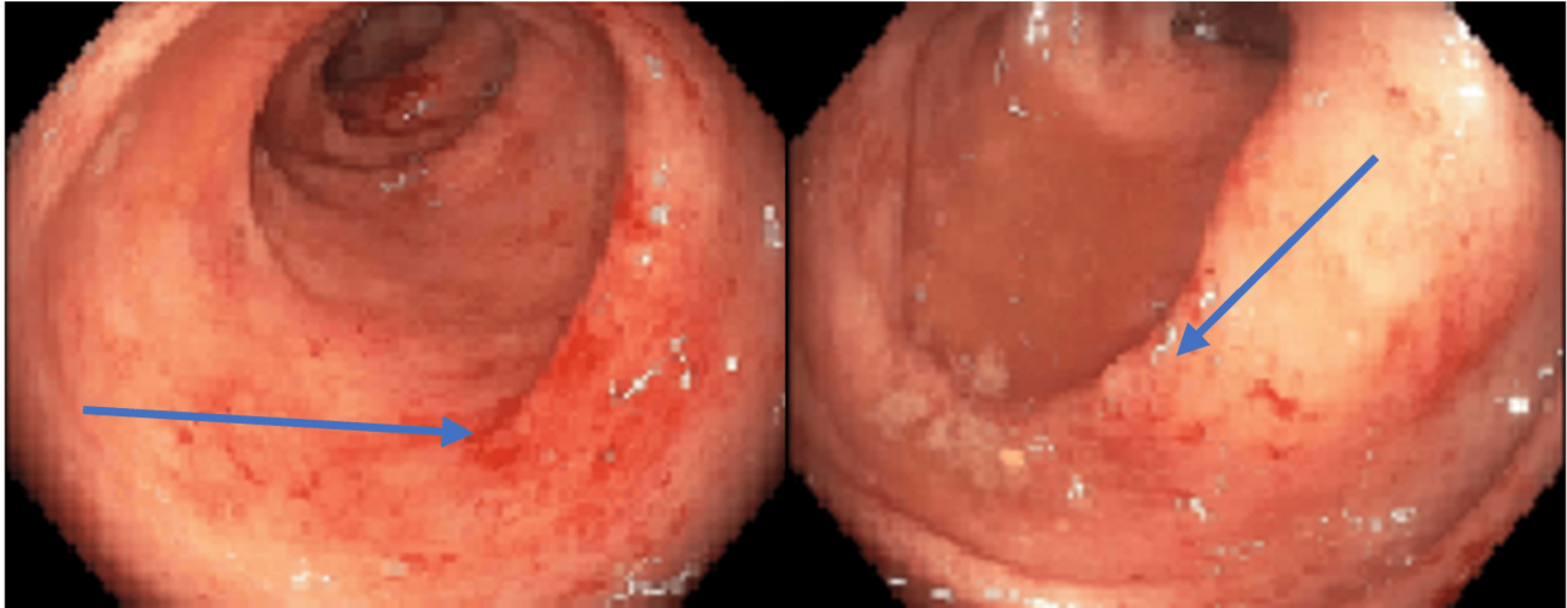
Colonoscopy showing colitis of the descending colon (blue arrow). Image obtained with the patient’s expressed consent.

**Figure 5:**
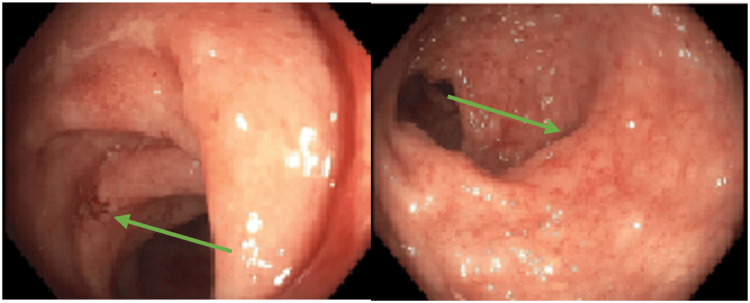
Colonoscopy showing colitis (green arrows) in the sigmoid colon proximal to the rectum. Images of the rectum were not obtained. Image obtained with the patient’s expressed consent.

**Figure 6:**
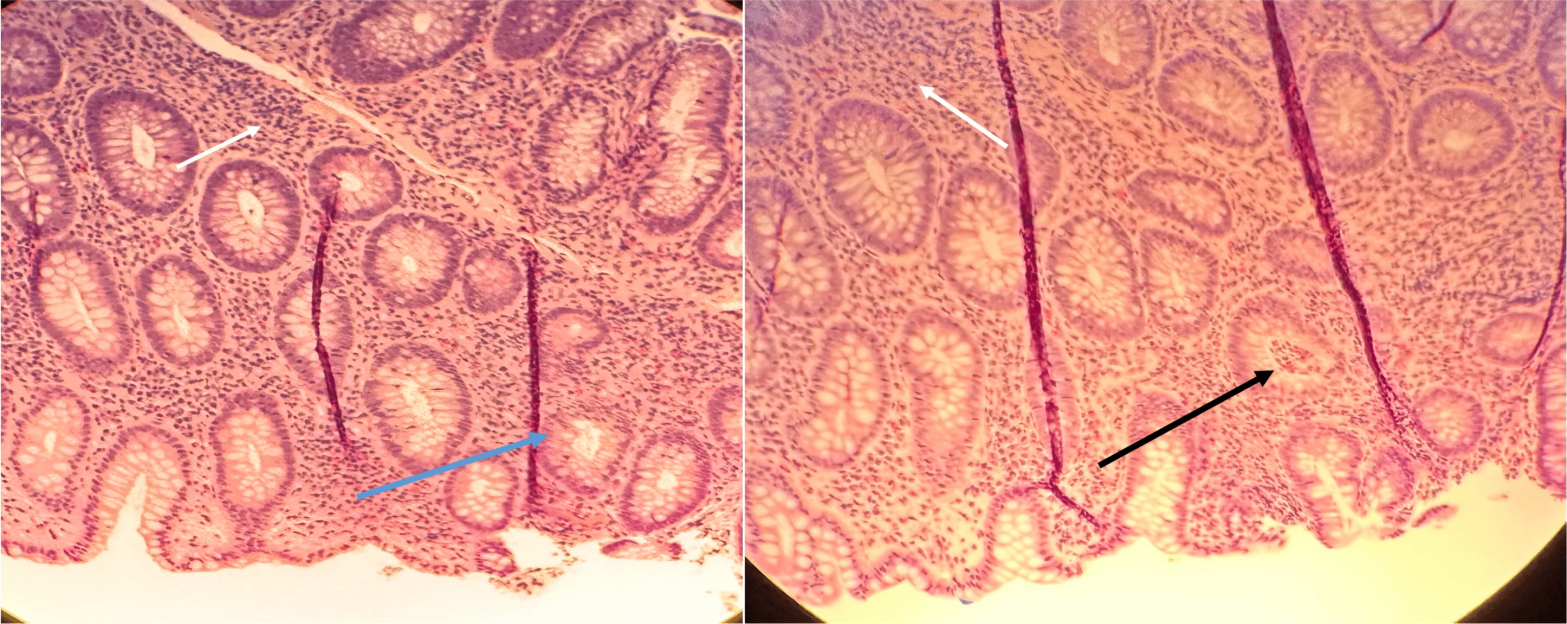
Biopsy of the descending colon showing extensive neutrophilic infiltration of the lamina propria (white arrows), early neutrophilic infiltration of the crypts (blue arrow) suggestive of cryptitis, and evidence of a crypt abscess (black arrow). Image obtained with the patient’s expressed consent.

**Figure 7:**
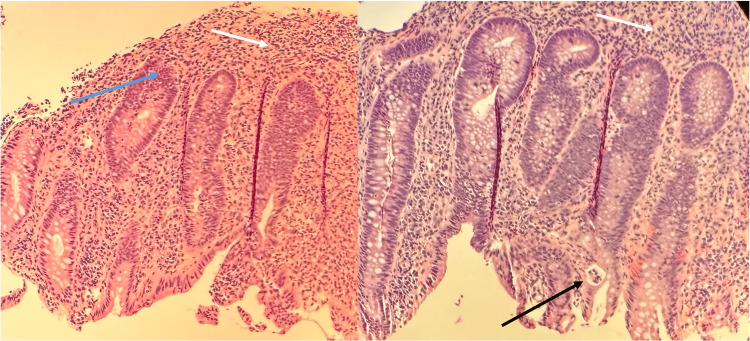
Biopsy of the rectum showing neutrophilic infiltrates in the lamina propria (white arrows), early neutrophilic infiltration into the crypts (blue arrow), and a crypt abscess (black arrow). Image obtained with the patient’s expressed consent.

Colonoscopy revealed significant mucosal inflammation in the left descending colon (Figure 4) and sigmoid colon proximal to the rectum (Figure 5), suggestive of colitis. Rectal mucosa images were not obtained during the procedure. Histopathological staining of the descending colon biopsy showed extensive neutrophilic infiltration in the lamina propria and crypts, along with crypt abscesses (Figure 6). Rectal biopsies also showed neutrophilic infiltration and crypt abscesses, indicative of proctitis (Figure 7).

These findings were suggestive of acute colitis secondary to chronic NSAID use; however, the gastroenterology team provided a working diagnosis of inflammatory bowel disease (IBD). Once hemodynamically stable, the patient was discharged on oral prednisone and mesalamine for active IBD and was advised to discontinue NSAID use. He was instructed to return in six weeks for a repeat colonoscopy, but he ultimately failed to follow up and was lost to follow-up. 

## Discussion

Incidence and prevalence of acute colitis secondary to chronic NSAID use

The National Institutes of Health has concluded that, per year in the United States, there is a prevalence of 0.067%, or 67 in 100,000, for upper GI bleeds, and a prevalence of 0.036%, or 36 in 100,000, for lower GI bleeds [4]. In patients with chronic NSAID use, a positive association has been found with serious GI-related complications such as bleeding or perforations. A recent statistical meta-analysis conducted in 2023 by Haider et al. found that, of 133,149 chronic NSAID users assessed in 2021, there was a prevalence of various GI-related pathologies: 163 patients were found to have microscopic colitis (0.12%), 640 patients had ulcerative colitis (0.48%), and 847 patients had Crohn’s disease (0.64%) [7]. Comparatively, among patients who did not have chronic NSAID use, the prevalence of these pathologies was significantly lower, with values of 0.09% for microscopic colitis, 0.35% for ulcerative colitis, and 0.58% for Crohn’s disease. These findings support the theory that chronic NSAID users are more likely to develop GI-related complications when compared to non-NSAID users. Moreover, it was also found that patients above the age of 40 had an added risk of developing IBD, with an incidence of 21-36% for ulcerative colitis and 40-50% for Crohn’s disease [5,6].

NSAID mechanism of action

While a detailed literature review of the physicochemical makeup of NSAIDs is beyond the purview of this case report, it is noted that the side effects of NSAIDs are directly related to their interaction with the COX enzyme pathways. As highlighted previously, the COX enzyme exists in two distinct isoforms: COX-1 and COX-2. COX-1 is expressed throughout the body in multiple tissue types, with functions including gastric cytoprotection, platelet aggregation, and maintenance of renal function. COX-2 is primarily involved in inflammatory response pathways and is highly regulated in response to luminal aggressors. NSAIDs primarily function by inhibiting COX enzymes and their associated pathways, thereby disrupting the production of arachidonic acid derivatives, such as prostaglandins, prostacyclins, and thromboxanes, that mediate inflammation-related symptoms. Thus, the function of COX enzymes ranges from sustaining hemostatic function to mediating physiological responses to noxious stimuli [7].

COX-2 plays a critical role in GI cytoprotection through the production of prostaglandins, specifically prostaglandin E2 and prostaglandin I2. These are the main endogenous autocrine factors that aid in the inhibition of acid secretion, histamine-stimulated parietal cell function, gastrin-stimulated histamine release from gastric mucosal cells, and the overall enhancement of GI mucosal defense [8]. Prostaglandins have also been shown to regulate mucosal blood flow via vasodilation in the setting of disrupted epithelial barrier function, thereby protecting the surrounding submucosa by neutralizing the effects of acid or other harmful insults. Therefore, prostaglandin synthesis is vitally important in maintaining the equilibrium of the GI mucosal layer. Accordingly, chronic NSAID use can lead to sustained inhibition of this protective mechanism, thereby increasing the risk of significant mucosal damage in the GI tract.

Pathogenesis of NSAID-induced GI pathologies

Cells in the GI tract are known to possess innate capabilities for mucus production and secretion. This mucus functions as a lubricant to aid in digestion and provides a protective layer between the surface epithelium and potentially harmful luminal contents such as acid, bacteria, or cytotoxic substances. While chronic NSAID use has been associated with the development of GI pathologies, a study conducted in 2017 showed that this factor is not the sole cause of pathological processes. Bjarnason et al. concluded that chronic NSAID use, in collaboration with luminal aggressors, leads to the formation of ulcers, ulcerative bleeds, and GI mucosal erosion [9]. An example of a luminal aggressor would be a substance that disturbs the mucus layer or its production, thereby providing an access point for harmful luminal contents into the submucosal layer. Given this premise, there are two proposed pathophysiological mechanisms for NSAID-induced acute colitis. 

The first proposed mechanism, considered the lesser of the two, presupposes that with oral intake of NSAIDs, there is a highly localized concentration present in the GI lumen. This, in conjunction with bile secretion, has been shown to increase the permeability of the intestinal mucosa. This theory also suggests that NSAIDs may be introduced into enterohepatic circulation, and that repeated exposure leads to NSAID toxicity and GI mucosal damage. Two separate studies explored this theory and found a statistically significant positive correlation between NSAIDs undergoing enterohepatic circulation and increased epithelial permeability and intestinal ulceration, observed in both human and animal subjects [9,10]. 

The second proposed mechanism, which is relatively more widely accepted, consists of two subsequent processes. First, localized mucosal injury occurs with an increase in leukocyte adherence to the vascular endothelium, which disrupts the cellular repair mechanism. Second, the direct toxic effects of NSAIDs inhibit epithelial repair in the setting of mucosal damage. Initially, there is localized mucosal injury caused by NSAID inhibition of COX, which prevents the synthesis of prostaglandins. This compromises the cytoprotective layer of the surface gastric epithelium, leading to gradual disruption and cumulative damage to the mucosal barrier. Continuous damage ultimately results in macroscopic and often symptomatic injuries, including epithelial erythema, subepithelial hemorrhages, or mucosal ulcerations. Additionally, there is significant evidence of increased leukocyte adhesion (typically neutrophils) to the vascular endothelium of the GI microcirculation, which further disrupts gastric mucosal blood flow and impairs the healing process [11]. 

In addition, NSAIDs are also proposed to have direct toxic effects on the epithelial repair process. A study conducted in 2008 showed that prostaglandin synthesis is upregulated in response to mucosal damage, promoting vasodilation and increased local blood flow to support rapid epithelial repair, thereby preventing damage to the deeper submucosal and basal layers. With chronic NSAID use, the reduction in prostaglandin synthesis negates this natural upregulation, resulting in a lack of localized vasodilation. This ultimately leads to delayed healing and increased vulnerability to further epithelial damage [11]. These cumulative effects of chronic NSAID use lead to macroscopic and microscopic GI injuries, ultimately resulting in clinically significant and potentially debilitating pathologies.

Clinical manifestations and symptomatic presentations

Unlike other pathological processes, NSAID-induced colitis results from cumulative, gradual damage to the GI mucosa that requires consistent and continuous insults. It is more likely to be seen in patients with chronic NSAID use, who rely on the anti-inflammatory properties of these medications for symptomatic relief in chronic inflammatory pathologies (such as rheumatic conditions or chronic pain). For this reason, clinicians commonly prescribe a gastro-protective agent to be taken concurrently with daily NSAID use in an attempt to prevent mucosal injury. In the general population, where NSAID therapy is used sporadically, it is unlikely to see sustained GI mucosal damage and its symptomatic presentation. 

As with most conditions, symptoms vary between patients. Generally, however, the initial symptomatic presentation of NSAID-induced acute colitis is often related to iron deficiency anemia secondary to occult GI bleeding. These symptoms include, but are not limited to, generalized fatigue and malaise, conjunctival and mucosal pallor, non-specific abdominal pain, or blood per rectum. Further work-up often reveals abnormal hemoglobin and iron values, as well as a positive stool guaiac test. This patient presented with non-specific symptoms related to anemia, such as generalized fatigue and malaise; however, he did not show any signs of conjunctival or mucosal pallor. Routine blood work revealed a decreased hemoglobin level of 7.9 g/dL (N: 13.2-16.6 g/dL), which prompted further questioning that ultimately revealed chronically ongoing hematochezia and melena. Subsequent investigations also showed a positive stool guaiac test and maroon-colored stool in the rectal vault, indicating ongoing GI bleeding. 

It is, of course, surprising that the patient failed to disclose ongoing bloody stool during his initial annual visit, as this is commonly recognized as an abnormal symptom. However, this highlights the importance of thorough history-taking during routine annual examinations. It also underscores the necessity of cautioning patients about the adverse effects associated with chronic NSAID use. Given the widespread availability of these medications and their over-the-counter accessibility, it is imperative that patients suffering from chronic inflammation or long-term pain be advised to avoid NSAIDs, or to use them only under the direct guidance of a supervising clinician.

Diagnostics 

The diagnostic criteria for NSAID-induced acute colitis follow a similar pattern to that of GI bleeds with an unknown etiology. Initial assessment should focus on the hemodynamic stability of the patient. If the patient requires hospital admission for hemodynamic support, resuscitative efforts take priority to stabilize the patient; these include intravenous fluid therapy or blood transfusions if warranted. Upon achieving hemodynamic stability, further work-up to assess the source of the bleed can be conducted. This often begins with endoscopic investigations such as esophagogastroduodenoscopy and/or colonoscopy, with biopsies taken as needed; exploration of both the upper and lower GI anatomy ensures comprehensive evaluation of potential GI-related pathologies.

In patients with increased risk factors, it is not uncommon to see the presence of diaphragmatic strictures, which are pathognomonic for NSAID-induced acute colitis. Diaphragmatic strictures, caused by mucosal injury, are thin, concentric lesions that decrease the intra-luminal diameter, resulting in narrowing of the lumen. These can be found throughout the GI tract; however, they are more commonly observed near the ileocecal junction. Histopathological examination of these lesions shows submucosal fibrosis secondary to chronic inflammation, with an overlying layer of surface epithelium [12]. Notably, if the lesion is found proximal to the gastroesophageal junction, the pathology is often unrelated to NSAID use. 

If there is a high degree of clinical suspicion for the presence of strictures, it is imperative that clinicians also obtain biopsy samples from non-stricture epithelium during endoscopic evaluation. These samples allow for better visualization and assessment of other potential differential diagnoses, such as infectious colitis, irritable bowel syndrome, ischemic colitis, radiation colitis, and vasculitides. For example, if cobble-stoning or crypt abscesses are observed, this would point the clinician towards IBD [13]. In this case, the patient did have significant mucosal inflammation, neutrophilic infiltrates, and crypt abscesses observed on histochemical staining of colonic biopsy samples. These are general findings seen in both acute colitis and IBD. However, with a relevant history of chronic NSAID use and the absence of any family history of GI pathologies, it is more likely that these findings are related to NSAID-induced acute colitis. For this reason, a follow-up colonoscopy is typically scheduled six weeks after hospital discharge; the persistence of these findings on follow-up would further suggest IBD, whereas their absence would indicate a resolved case of NSAID-induced acute colitis. In this particular case, the patient was lost to follow-up and a repeat colonoscopy was not performed. While it is not possible to confirm whether this patient suffered from IBD, it is reasonable and logical to presume that the lack of follow-up may be indicative of symptomatic resolution, thus supporting the diagnosis of NSAID-induced acute colitis. This further highlights the importance of patient education regarding follow-up testing and its relevance to diagnosis and management plans.

Management

The mainstay treatment of NSAID-induced acute colitis involves the cessation of the inciting insult, NSAIDs. In the absence of other underlying GI diseases, discontinuation of NSAID therapy often leads to complete symptomatic resolution. Removing the inhibitor of the body’s natural repair processes allows prostaglandins to resume normal function and help re-form the GI mucosal layer. Patients who do not have any imaging or endoscopic evidence of strictures are typically instructed to undergo a repeat colonoscopy 6-8 weeks after discontinuing NSAID use. This is done to ensure mucosal healing and confirm full resolution of the colitis. 

In patients found to have strictures, a different management approach may be necessary. Depending on the extent of the lesion, treatment may involve through-the-scope balloon dilation to expand the diameter of the lumen to a near-normal state [14]. In more severe cases where balloon dilation is not feasible, patients may require intraoperative enteroscopy with wide-margin bowel resection to ensure complete removal of the obstructive lesion [15,16]. This is done to prevent progression to bowel obstruction. The decision on the appropriate stricture management approach is case-specific and depends on several factors, including the number and location of strictures and the risk-benefit profile for each individual patient.

Etiological possibilities in this case

The complications associated with NSAID-induced acute colitis are closely linked to comorbidities and continued NSAID use. In this case, the patient’s risk was stratified as low to moderate, primarily due to his daily NSAID use for nearly three years prior to admission. It is plausible that the patient may have an unknown underlying etiology, genetic or environmental, that could predispose him to the development of IBD later in life. However, even in such a scenario, prolonged NSAID use would likely have contributed to an earlier onset of colitis. 

Throughout the hospital stay, providers remained conflicted about the etiology of the patient’s symptoms and investigative findings. Some clinicians believed this to be a case of early-onset ulcerative colitis, while others were convinced it was related to chronic NSAID use. It is important to note that both conditions can present similarly, with blood in the stool, leukocyte infiltrates on histopathological staining, and crypt abscesses. However, considering the patient’s history and overall clinical picture, NSAID-induced acute colitis appears to be the more likely diagnosis in this case. A repeat colonoscopy on follow-up would have confirmed this diagnosis by demonstrating resolution of the presenting findings. However, as the patient was lost to follow-up, this was unfortunately not possible.

## Conclusions

This case highlights the adverse effects associated with chronic NSAID use, as well as the diagnostic challenges in distinguishing NSAID-induced colitis from inflammatory bowel disease, given their overlapping clinical and histopathological features. Although the patient was diagnosed with inflammatory bowel disease, the absence of a strong family history and the presence of chronic NSAID use raise the possibility of drug-induced colitis. This underscores the importance of obtaining a detailed medication history and ensuring close outpatient follow-up. In this case, reinforcement of NSAID cessation and consideration of a repeat colonic biopsy are warranted to clarify the diagnosis and guide long-term management. Ultimately, clinicians must maintain a high index of suspicion for medication-related gastrointestinal injury to avoid misdiagnosis and to deliver timely, appropriate care.
